# Supplementary material for: Inhibition of complement activation, myeloperoxidase, NET formation and oxidant activity by PIC1 peptide variants
Source: PLoS One. 2019 Dec 31;14(12):e0226875. doi: 10.1371/journal.pone.0226875 (PMC6938345; doi:10.1371/journal.pone.0226875)
Supplement: Attachment — Submitted filename: PONE_letter_20191106_response_20191111.docx [file pone.0226875.s002.docx]

PONE-D-19-26436
Inhibition of complement activation, myeloperoxidase, NET formation and oxidant activity by PIC1 peptide variants.

Dear Dr. Li:

Please find below our response to reviewers’ comments.

*Editor: Please confirm that this does not alter your adherence to all PLOS ONE policies on sharing data and materials, by including the following statement: "This does not alter our adherence to  PLOS ONE policies on sharing data and materials.” Please include your updated Competing Interests statement in your cover letter; we will change the online submission form on your behalf.*

RESPONSE: We have added the requested statement to the cover letter.

**Comments to the Author**
5. Review Comments to the Author

*Reviewer #1: This manuscript tested sixteen sarcosine substitution variants for solubility in water. Aqueous soluble variants were then tested in standard complement, myeloperoxidase, NET formation and antioxidant capacity assays. However, we can't see the significance of doing this study, either clinical significance or the structural biological significance.*

RESPONSE: We understand the Reviewer’s concern and have addressed this by expanding the Discussion section with additional details about the clinical significance. (See response to reviewer #3).

Reviewer #2: *revise the manuscript as their are some missed letters in some words.*

RESPONSE: We performed a spell check and corrected identified deficiencies.

*Pooled Normal Human Serum (NHS) (add their preparation)*

RESPONSE: We have added the requested description of NHS preparation to the Methods section.

*authors should give a list for abbreviations*

RESPONSE: We have added a list of abbreviations, although this is not requested in the PLoS One submission guidelines.

*Figure ligands were written in the result section: they should be separated at the end of the manuscript.*

RESPONSE: We have followed PLoS One submission guidelines which state, “Figure captions must be inserted in the text of the manuscript, immediately following the paragraph in which the figure is first cited.”

Reviewer #3:
*The potential theraputic uses of the peptides based on their described in vitro activity is not discussed by the authors and this may be appropriate for this paper, but are obvious.*
RESPONSE: We agree with the Reviewer’s note and have added additional detail to the Discussion about potential therapeutic uses.
